# Association of Psychologic and Nonpsychologic Factors With Primary Dysmenorrhea

**DOI:** 10.5812/ircmj.16307

**Published:** 2014-08-05

**Authors:** Mahbobeh Faramarzi, Hajar Salmalian

**Affiliations:** 1 Fatemeh Zahra Infertility and Reproductive Health Research Center, Department of Midwifery, Babol University of Medical Sciences, Babol, IR Iran

**Keywords:** Dysmenorrhea, Alexithymia, Personality

## Abstract

**Background:**

Primary dysmenorrhea seems to be one the most common gynecologic condition in women of childbearing age.

**Objectives:**

The aim of this research was to evaluate psychologic and nonpsychologic risk factors of primary dysmenorrhea.

**Materials and Methods:**

A cross-sectional study was conducted on medical sciences students of Babol University of Medical Sciences. In this study, 180 females with dysmenorrhea and 180 females without dysmenorrhea were enrolled. Psychological risk factors were evaluated in four domains including affect, social support, personality, and alexithymia. Four questionnaires were used to assessed aforementioned domains, namely, Social Support Questionnaire (SSQ), depression, anxiety, stress (DAS-21), 20-item Toronto Alexithymia Scale (TAS-20), and NEO-Five Factor Inventory of Personality (NEO-FFI). In addition, nonpsychologic factors were evaluated in three domains including demographic characteristics, habits, and gynecologic factors. Data were analyzed using the χ2 test and multiple logistic regression analysis.

**Results:**

The strongest predictor of primary dysmenorrhea was low social support (OR = 4.25; 95% CI, 2.43-7.41). Risk of dysmenorrhea was approximately 3.3 times higher in women with alexithymia (OR = 3.26; 95% CI, 1.88-5.62), 3.1 times higher in women with menstrual bleeding duration ≥ 7 days (OR = 3.06; 95% CI, 1.73-5.41), 2.5 times higher in women with a neurotic character (OR = 2.53; 95% CI, 1.42-4.50), 2.4 times higher in women with a family history of dysmenorrhea (OR = 2.43; 95% CI, 1.42-4.50), and twice higher in women with high caffeine intake (OR = 1.97; 95% CI, 1.09-3.59).

**Conclusions:**

Low social support, alexithymia, neuroticism trait, long menstrual bleeding, family history of dysmenorrhea, and high-caffeine diet are important risk factors for women with primary dysmenorrhea. This study recommended considering psychologic factors as an adjuvant to medical risks in evaluation and treatment of primary dysmenorrhea.

## 1. Background

Dysmenorrhea is defined by uterine muscle spasms beginning shortly before or at the onset of menstruation (1, 2). It is categorized in two types: primary and secondary. Primary dysmenorrhea is recognized by painful menstrual cramps without any organic pathology (3). It usually occurs three years after menarche (4). According to studies, there is a wide variation in the prevalence of dysmenorrhea ranging from 28% to 77.7% worldwide (5, 6). In a survey from Iran, the prevalence of 63.5% was reported for dysmenorrhea in medical students (7). In addition, dysmenorrhea is a common cause of absenteeism from work and classes by the female students (8). Therefore, it is a disabling condition among women of childbearing age (9).

Dysmenorrhea should be considered as a part of the medically unexplained syndromes and be viewed as a multifactorial disorder (10). Studies on the dysmenorrhea have shown that many factors are associated with this syndrome. These factors include a younger age, low body mass index (BMI), smoking, early menarche, prolonged or aberrant menstrual flow, pelvic infections, previous sterilization, genetic influence, a history of sexual abuse (11), high caffeine intake (12), and breakfast consumption (13).

Although psychosocial risk factors of primary dysmenorrhea have not been studied extensively, there is growing evidence of a psychologic etiology. Previous studies reported that women with dysmenorrhea tend to be more preoccupied with bodily sensations, tend to express greater negative attitudes toward illness, and have more negative attitude toward menstruation than do other women (14). Ambresin et al. found that patients with severe dysmenorrhea not only show a different proﬁle from their peers in terms of their mental health, but are also more dissatisﬁed with their body appearance (15). Some psychologic factors such as high emotional disturbance, and psychologic symptoms were found to be associated with higher rates of dysmenorrhea. A study showed that dysmenorrhea intensity increased with the severity of depression, anxiety, and somatic complaints (10). Researches mentioned that menstrual irregularities can be used as an indicator of psychologic social adjustment disorder in 13 to 19-year-old girls during the early years after menarche (16).

There is little information on the psychologic risk factors for primary dysmenorrhea. Personality trait, affect, social support, and alexithymia might influence women with primary dysmenorrhea. A previous study showed that social support in women with dysmenorrhea was less than women without dysmenorrhea (17). In addition, a report emphasized that there is an association between personality trait (neuroticism) and menstrual pain (18). The aim of this study was to compare university students with dysmenorrhea and without dysmenorrhea with regard to four domains: demographic, habitual, gynecologic, and psychologic factors. Measured psychologic factors included social support, affect (depression, anxiety, and stress), personality traits (neuroticism, extraversion, openness to experience, agreeableness, and conscientiousness), and alexithymia.

## 2. Objectives

The aim of this research was to evaluate psychologic and nonpsychologic risk factors of primary dysmenorrhea.

## 3. Materials and Methods

This cross-sectional study was conducted between November 2012 and March 2013 on medical sciences students of Babol University of Medical Sciences (Babol City, north of Iran). We enrolled 180 woman with and 180 women without dysmenorrhea. Inclusion criteria were primary dysmenorrhea, which had started up to two years of menarche, no history of pelvic or abdominal surgery, and willingness to participate in the study. Women with secondary dysmenorrhea were excluded. The following criteria were used to define dysmenorrhea: beginning of pain within six to 12 hours of menstruation, lower abdominal pain associated with beginning of menstruation and lasting for eight to 72 hour, and low back pain during menstruation (19). The students with mild to severe primary dysmenorrhea were included in the study. 

The dysmenorrhea pain was measured in each participant by a verbal multidimensional scoring system based on the degree of pain, restriction, and activities; the scientific validity and reliability of this scoring system was confirmed previously. The system consists of four scores. The absence of dysmenorrhea was defined as zero, which meant there is no interference with daily activities. Grade one was mild menstrual pain that rarely interfered with daily activities, mild systemic symptoms, and very little need to analgesia. Grade two was the presence of moderate pain and possible disruption of daily activities, but there was no need to miss school or work. Grade three was severe pain to the degree that the person was not able to perform daily activities and severe systemic symptoms were observed (20). 

Ethical approval was granted by the Medical Education Ethics Committee at Babol University of Medical Sciences (ID: 3150; date: February 28, 2010). According the ethical research, each student had to sign an informed consent and her information had to be confidential. After permission of the deans of faculty, a multistage cluster sampling was employed to recruit the medical sciences student based on their fields of study and the years of attending university. We estimated the sample size by the following formula:

N = (z_1_ - α/2 + z_1 _- β)^2^ (p_1_q_1_ + p2q2)/(p_1_ - p_2_)^2^

In this formula, p_1_ represent the proportion of physical and psychologic health in dysmenorrhea group and q_1_ the proportion of the population with dysmenorrhea with the physical and psychologic problems. In addition, p_2_ and q_2_ determined the aforementioned proportions in the group without dysmenorrhea. 

Considering above formula with z_1_ - α/2 = 1.96, z_1 _- β = 0.84 (with 95% CI and power of 80%), p_1_ = 0.40, and p_2_ = 0.55, the sample size was estimated at 170 in each group; however, we considered 360 subjects to compensate probable samples drop-out during study.

Medical sciences students consisted of medicine, midwifery, nursing, physiotherapy, health, and laboratory sciences. According to field and academic years of the student, there were 30 classes. Fifteen single students with 18 to 24 years of age were randomly selected from the students list of each class. Then 450 female students were invited to participate in the study. Members of the research team gave a brief explanation to the students regarding the purpose of the study. Finally, 402 students agreed to participate and were asked to complete five questionnaires. Afterwards, 37 students who answered incompletely or did not respond the questionnaires as well as five students with suspected secondary dysmenorrhea were excluded. Finally, 360 subjects (180 with dysmenorrhea, 180 without dysmenorrhea) were enrolled into the final analysis. The five questionnaires used in this study concerned psychologic and nonpsychologic data. The questionnaire included three parts: demographic, habitual, and gynecologic factors. Psychologic risk factors were evaluated in four domains including affect (depression, anxiety, and stress), social support, personality traits, and alexithymia by means of depression, anxiety, stress (DAS-21), Social Support Questionnaires (SSQ), NEO-Five Factor Inventory of Personality (NEO-FFI), and 20-item Toronto Alexithymia Scale (TAS-20), respectively. These questionnaires took only 60 minutes to complete.

### 3.1. Scales and Measurement

#### 3.1.1. Nonpsychologic Questionnaire

This questionnaire was prepared with reference to previous available researches in the literature (11, 12, 20) and included three parts. Firs part concerned “demographic factors” and included age, socioeconomic status (SES), number of family members, and BMI. BMI was classiﬁed as normal weight (18.0-24.9 kg/m^2^), overweight/obese (> 25.0 kg/m^2^), and underweight (< 18.0 kg/m^2^) (21). Second part concerned “gynecologic factors” and included age at menarche, family history of dysmenorrhea, menstrual bleeding duration, menstrual interval, and presence of premenstrual syndrome. The presence of dysmenorrhea in a student’s mother or sister was considered as a positive family history. Menstrual bleedings in equal intervals between 21 and 35 days were considered as normal interval. The menstruation intervals of shorter than 21 days or longer than 35 days were considered as short and long, respectively. Menstruation of shorter than two days was defined as short, between two and six days as normal, and longer than six days as long (22). Third part concerned “habitual factors” and included caffeine intake, having breakfast, and exercise. Caffeine intake was defined as excessive if consumption of caffeinated soft drinks, coffee, decaffeinated coffee, tea, chocolate milk, and chocolate bars in the daily diet was self-reported as ≥ 300 mg/day and as minimum to moderate if the daily intake of the listed items was < 300 mg/day. Having enough exercisers was defined as participating in physical activity ≥ 3 times per week, with each session lasting more than ten minutes (23, 24). Having breakfast was classified as normal if participant had breakfast one to six times per week and as low if she had breakfast less than once a week (13).

#### 3.1.2. Depression, Anxiety, Stress Scale 

The DAS-21 is a 21-item self-report questionnaire, which is designed to measure a range of common symptoms of depression, anxiety, and stress. This instrument contains three subscales that cover depression (7 items), anxiety (7 items), and stress (7 items). Each item is scored from zero (i.e. not at all) to three (i.e. very much). Therefore, total scores of each subscale ranges from zero to 21. The DAS-21 is the short form version of DAS, the original and long form with 42 items; therefore, the final score of each subscale needs to be multiplied by two. The following cutoff score is used to assess the presence of the symptoms: depression ≥ 10, anxiety ≥ 8, and stress ≥ 15 (25). A valid Farsi version of DAS-21 was used in the study (26).

#### 3.1.3. The Scale of Perceived Social Support

Social Support Questionnaires (SSQ), which was developed by Fleming et al. has 25 items and five subscales: perceived social support by the family (7 items), by friends (3 items), by the neighbors (4 items), and by the public (6 items), and the notion or opinion about the support (5 items) (27). The coefficient validity of this scale was calculated at 0.68 in Iranian population by using Cronbach’s alpha coefficient (28). In the current research, the validity of SSQ by the assessment of Cronbach’s alpha coefficient was calculated at 0.72. We categorized total scores of social support based on the distribution of the scores in the student population. We applied cutoff scores of ≤ 11 for low and ≥ 12 for moderate/high social support. In addition, scores of 12 to 16 were defined as moderate and 17 to 25 as high social support.

#### 3.1.4. Toronto Alexithymia Scale 

Alexithymia is defined as difficulty in identifying feelings and distinguishing between feelings and the bodily sensations of emotional arousal, difficulty in describing feelings to other people, and constricted imagined processes (29). Alexithymia was evaluated with the self-reported 20-item TAS-20, which is a widely-used and well-validated measure of alexithymia. This instrument contains 20 items and three subscales that cover difficulty in identifying feelings (7 items), the difficulty in describing feelings (5 items), and externally-oriented thinking (8 items). The scores are on a Five-point Likert scale which is scored from one through five. Therefore, total score of TAS-20 ranges from 20 to 100 (30). We used cutoff scores of ≤ 50 for non-alexithymia and ≥ 51 for alexithymia (29). A validated version of the TAS-20 for Iranian population was used in this research (31). In the current study, the validity of TAS-20 by the Cronbach’s alpha coefficient was calculated at 0.85.

#### 3.1.5. NEO-Five Factor Inventory 

The NEO-FFI was developed by Costa and McCrae to describe human personality, which is a 60-item questionnaire that measures the five personality traits, namely, neuroticism, extraversion, openness, agreeableness, and conscientiousness. Internal consistency (coefficient alpha) of the five scales was calculated at 0.73 (32). Neuroticism is sometimes called emotional instability. It is the sentiment to experience negative emotions such as anger, anxiety, or depression. Extraversion is characterized by positive emotions and the sentiment to seek out stimulation and the company of others. Openness is a general understanding for art, emotion, adventure, unusual ideas, imagination, curiosity, and variety of experience. Agreeableness is a sentiment to be compassionate and cooperative rather than suspicious and antagonistic towards others. Conscientiousness is a sentiment to show self-discipline, act dutifully, and aim for achievement against measures or outside expectations (33). These scales were developed by taking the 12 items with the highest positive or negative factor. Items of scale require responses on a five-point scale (1 through 5) from strongly disagree to strongly agree (32). Then, crude score of all of the subscales ranges from 12 to 60. We changed crude scores to T scores (mean 50, SD 10). According to T scores, cutoff for all subscales was considered as follows: ≥ 56, high; 45 to 55, moderate; and ≤ 44, low (34). A valid Farsi version of the NEO-FFI was used in this study (35).

### 3.2. Statistical Analysis

All variables were at first ﬁtted as categorical variables, and tests for non-linearity were performed as appropriate. Continuous factors, such as age, and family number were categorized based on the distribution of scores. Dysmenorrhea was introduced to statistical software as a dependent variable with a binary outcome (absent/present).

The analysis was performed using Chi square for the univariate association between dysmenorrhea and all the 20 psychologic and nonpsychologic factors including age, SES, number of family members, BMI, age at menarche, family history of dysmenorrhea, menstrual cycle duration, menstrual bleeding duration, caffeine intake, having breakfast, exercise, social support, alexithymia, depression, anxiety, stress, neuroticism, extroversion, openness to experience, and agreeableness. Then, each of psychologic and nonpsychologic factors with detected significant association at univariate analysis (P < 0.05) was included in a multiple logistic regression model. In data analysis, we applied PASW 18 (IBM, Armonk, NY, USA). Odds ratios and 95% conﬁdence intervals were presented for the main associations. A P value < 0.05 was considered statistically significant.

## 4. Results

The mean age of the participants was 20.41 ± 1.62 years (range, 17-25). With regard to age group, 58.3% of the students were 20 years old or younger. Poor SES was reported by 66.4% of students. The number of family members was ≤ 5 in 85.0% of students. Characteristics of students regarding the status of dysmenorrhea are shown in the Table 1. There was no difference between dysmenorrhea and non-dysmenorrhea group with regard to demographic variables (age, marital status, SES, and BMI), except for being the first child of the family (P < 0.001).

**Table 1.** Nonpsychologic Factors of the Study Population With Respect to Dysmenorrhea Status ^a^

Variables	Dysmenorrhea ^b^			Univariate OR (95% CI)	P Value
	Yes	No	Total		
**Age**				1.25 (0.8-1.9)	0.333
< 20 y	70 (46.7)	80 (53.3)	150 (41.7)		
≥ 20 y	110 (52.4)	100 (47.6)	210 (58.3)		
**SES**				0.76 (0.49-1.1)	0.256
Middle/High	125 (52.3)	55 (45.5)	121 (33.6)		
Low	114 (47.7)	66 (54.5)	239 (66.4)		
**Number of Family Members **				0.64 (0.3-1.1)	0.184
> 6	32 (59.3)	22 (40.7)	54 (15.0)		
≤ 5	148 (48.4)	158 (51.6)	306 (85.0)		
**BMI**					0.667
Overweight/Obese	10 (5.6)	13 (7.2)	23 (6.4)		
Normal	157 (87.2)	155 (86.1)	312 (86.7)		
Low	13 (7.2)	12 (6.6)	25 (6.9)		
**Age at Menarche**				1.07 (0.6-1.7)	0.823
≤ 12 y	62 (51.2)	59 (48.8)	121 (33.6)		
> 12 y	118 (49.4)	121 (50.6)	239 (66.4)		
**Family History of Dysmenorrhea**				2.63 (1.7-4.0)	< 0.001
No	81 (45.0)	99 (55.0)	180 (50.0)		
Yes	123 (63.3)	57 (31.7)	180 (50.0)		
**Menstrual Cycle Duration**				0.61 (0.3-1.1)	0.162
Normal (21-35 d)	161 (51.6)	151 (48.4)	312 (86.7)		
Abnormal (< 21 d or ≥ 35 d)	19 (39.6)	29 (60.4)	48 (13.3)		
**Menstrual Bleeding Duration, d**				2.13 (1.4-3.4)	0.001
< 6	101 (43.2)	133 (56.8)	234 (65.0)		
≥ 7	79 (62.7)	47 (37.3)	126 (35.0)		
**Caffeine Intake Level**				2.36 (1.4-3.8)	< 0.001
Low/Middle	116 (44.3)	146 (55.7)	262 (72.8)		
High	64 (65.3)	34 (34.7)	98 (27.2)		
**Having Breakfast **				1.52 (0.7-3.2)	0.177
Low/Middle	161 (49.1)	167 (50.9)	328 (91.1)		
High	19 (59.4)	13 (40.6)	32 (8.9)		
**Exercise**					0.123
Not Enough	145 (49.1)	150 (50.9)	295 (63.9)	1.	
Enough	35 (53.9)	30 (46.1)	65 (36.1)	32 (0.6-2.9)	

^a^ Abbreviations: OR, odds ratio; CI, confidence interval; SES, socioeconomic status; and BMI, body mass index.

^b^ Data are presented as No. (%).

Gynecologic factors of students with regard to the presence of dysmenorrhea are presented in Table 1. Familial history of dysmenorrhea was reported by 50.0% of students. The severity of dysmenorrhea was mild in 30.6%, moderate in 44.4%, and severe in 25%. The mean of menstrual bleeding duration was 6.38. ± 1.06 days with < 7 days in 65.0% of patients. Normal menstrual cycle duration was reported by 86.7% of students with the mean of 28.22 ± 3.77 days (range, 3-10). According to Table 1, there were significant differences between dysmenorrhea and non-dysmenorrhea group with regard to family history of dysmenorrhea and menstrual bleeding duration. There was no significant difference between two groups regarding age at menarche and menstrual cycle duration. Habitual factors of patients with and without dysmenorrhea are shown in Table 1.

There was no difference between groups with respect to having breakfast and exercise, except for tea and caffeine intake. Table 2 shows the psychologic factors of students with and without dysmenorrhea. Traits of alexithymia was seen in 178 students (49.4%). Univariate analysis showed that alexithymia was significantly more prevalent in students with dysmenorrhea than in those without dysmenorrhea (P < 0.001). Symptoms of depression, anxiety, and stress were reported in 34.4%, 40.6%, and 23.9% of students, respectively. In univariate analysis, students with dysmenorrhea had significantly more symptoms of depression, anxiety, and stress than those without dysmenorrhea (P < 0.01).

**Table 2.** Psychologic Factors of Study Population

Variables	Dysmenorrhea ^a^			Univariate, OR (95% CI)	P Value
	Yes	No	Total		
**Social Support**				3.54 (2.3-5.5)	< 0.001
Middle/High	57 (27.9)	147 (72.1)	204 (56.7)		
Low	105 (67.3)	51 (32.7)	156 (43.3)		
**Alexithymia**				3.45 (2.4-5.3)	< 0.001
No	64 (35.2)	118 (64.8)	182 (50.6)		
Yes	116 (62.5)	62 (34.8)	178 (49.4)		
**Depression**				2.60 (1.7-4.0)	< 0.001
No	99 (41.9)	137 (58.1)	236 (65.6)		
Yes	81 (65.3)	43 (34.7)	124 (34.4)		
**Anxiety**				2.11 (1.4-3.2)	< 0.001
No	91 (42.5)	123 (57.5)	214 (59.4)		
Yes	89 (61.0)	57 (39.0)	146 (40.6)		
**Stress**				2.11 (1.3-3.4)	0.002
No	125 (45.6)	149 (54.4)	274 (76.1)		
Yes	55 (64.0)	31 (36.0)	86 (23.9)		
**Neuroticism**				1.81 (1.1-2.9)	0.007
Low/Moderate	116 (45.7)	138 (54.3)	254 (70.6)		
High	64 (60.4)	42 (39.6)	106 (29.4)		
**Extroversion**				1.90 (1.0-3.3)	0.043
Low/Moderate	144 (47.5)	159 (52.5)	033 (84.2)		
High	36 (63.2)	21 (36.8)	57 (15.8)		
**Openness to Experience**				1.63 (0.8-3.2)	0.184
Low/Moderate	155 (48.6)	164 (51.6)	318 (88.3)		
High	25 (61.0)	16 (38.1)	42 (11.7)		
**Agreeableness**				1.73 (0.9-3.3)	0.069
Low/Moderate	154 (48.4)	129 (63.2)	204 (56.7)		
High	26 (69.1)	16 (32.7)	156 (43.3)		
**Conscientiousness**				0.65 (0.3-1.2)	0.124
Low/Moderate	156 (51.5)	147 (48.5)	303 (84.2)		
High	24 (42.1)	33 (57.9)	57 (15.8)		

^a^ Data are presented as No. (%).

High scores in personality traits of neuroticism, extraversion, openness, agreeableness, and conscientiousness were reported in 29.4%, 15.8%, 11.7%, 43.3%, and 15.8% of the students, respectively. Univariate analysis showed that neuroticism and extraversion were significantly more prevalent in students with dysmenorrhea (P < 0.05). There was no significant difference between students with and without dysmenorrhea with regard to openness, agreeableness, and conscientiousness. According to the bivariate analysis results (Tables 1 and 2), there was a signiﬁcant differences between students with and without dysmenorrhea in the following factors: family history of dysmenorrhea, menstrual bleeding duration ≥ 7 days, caffeine intake, alexithymia, low social support, depression, anxiety, and stress symptoms, and neurotic as well as extroversion personality. Then, logistic regression analysis performed with the above ten factors.

The results of multiple logistic regressions are shown in Table 3. According to this analysis, social support (OR = 4.25; 95% CI, 2.43-7.41), alexithymia (OR = 3.26; 95% CI, 1.88-5.62), menstrual bleeding duration ≥ 7 days (OR = 3.06; 95% CI, 1.73-5.41), neurotic personality (OR = 2.53; 95% CI, 1.42-4.50), a family history of dysmenorrhea (OR = 2.43; 95% CI, 1.42-4.50), and high caffeine intake (OR = 1.97; 95% CI, 1.09-3.59) had significant association with dysmenorrhea and were considered as its important risk factors.

**Table 3.** Risk Factors of Dysmenorrhea in Multiple Logistic Regression Analysis

Variables	β	SE	OR	95% CI	P
**Constant**	-3.310	0.395			< 0.001
**Family History of Dysmenorrhea **	0.869	0.266	2.38	1.41-4.01	0.001
**High Caffeine Intake**	0.682	0.304	1.97	1.09-3.59	0.025
**Menstrual Bleeding Duration ≥ 7 d**	1.121	0.290	3.06	1.73-5.41	0.000
**Presence of Premenstrual Syndrome**	0.175	0.341	1.19	0.66-2.14	0.559
**Low Social Support**	1.44	0.284	4.25	2.43-7.41	< 0.001
**Alexithymia Trait**	1.18	0.279	3.26	1.88-5.62	< 0.001
**Depression Symptom**	0.156	0.372	1.16	0.56-2.42	0.674
**Anxiety Symptom**	0.312	0.320	1.36	0.73-2.55	0.330
**Stress Symptom**	0.435	0.361	1.54	0.76-3.13	0.228
**Extroversion Trait**	0.632	0.371	1.88	0.90-3.88	0.088
**Neuroticism Trait**	0.869	0.266	2.53	1.42-4.50	0.002

## 5. Discussion

The results revealed that the strongest predictor of primary dysmenorrhea was low social support. In line with the present study and according to some researchers, women with dysmenorrhea reported inadequate social support than others (17). In contrast, some researches have emphasized that social functioning could not be affected by dysmenorrhea (20).

How we can explain the less adequate overall social support in students with dysmenorrhea? When this result was further investigated, the main difference was in the higher frequency of inadequate relationship with friends. It seems that the students with dysmenorrhea might have lees adequate social support, which is possibly characterized by the presence of unsatisfactory relationships. One possible explanation of the inadequate social support in students with dysmenorrhea might be related to high rate of alexithymia among this population. Many of patients with alexithymia have relationship disturbances (36); therefore, alexithymia seems to be an impairment mediator of social interaction. High levels of alexithymia is associated with limited social support, impaired interpersonal or social skills, fewer close relationships, preoccupation with somatic complaints, and depression (37).

Our finding showed that the second strongest predictor of primary dysmenorrhea was alexithymia. Higher levels of alexithymia in patients with chronic pain of dysmenorrhea were consistent with some studies that suggested women with chronic pain had significantly higher scores on the measure of alexithymia (38). Some of the possible underlying mechanisms have been proposed for association of alexithymia with the development of menstrual pain. First, individuals with high alexithymia have difficulties in recognizing their own physical and emotional symptoms, which may be linked to developing somatization pains (39). Second, individuals with high alexithymia have a limited ability to cope with stressful events (40). There is potential general hypersensitivity to both internal unpleasant sensations and externally induced pain in those with alexithymia (41). Finally, alexithymia has been shown to be associated with chronic pains by its effects on negative affect (42).

We found that the prevalence of primary dysmenorrhea was higher in the students whose menstrual bleeding duration was ≥ 7 days. This ﬁnding was consistent with the results showing that the risk of dysmenorrhea was higher in women with longer menstrual ﬂows (20). In addition, a meta-analysis study confirmed that heavy menstrual flow was a risk factor for dysmenorrhea (11).

According to our results, the prevalence of primary dysmenorrhea was significantly higher among women with neurotic personality trait. Some studies have indicated that women with neurotic personality trait have a higher risk of dysmenorrhea. Liang et al. concluded that in comparison to healthy controls, patients with dysmenorrhea had higher scores in neuroticism-anxiety characteristic (43). Nasyrova reported an association between dysmenorrhea and structure of neurotic disorders (44). Khalajinia et al. reported that frequency of introversion, and neuroticism was higher in the patients with dysmenorrhea than controls (45). The association between neuroticism and menstrual pain is probably due to influence of neuroticism characteristic on pain perception. Neuroticism is a vulnerability factor in which lowering the threshold of pain perceptions contributes to dysmenorrhea (18). In addition, high neuroticism is associated with the belief that pain is mysterious, aversive, and will last throughout of life (46).

According to our study, a family history of dysmenorrhea seems to be a risk factor for students with dysmenorrhea, which is consistent with other studies (20). Some research have suggested that the daughters of the mothers with menstrual complains also experience menstrual pain, which might be related to behavior that is learned from the mother (47).

The prevalence of dysmenorrhea was higher among students who had higher intakes of caffeine, which is compatible with other studies (12). It is unclear how high caffeine intake is related to dysmenorrhea; however, vasoconstricting actions of caffeine are implicated in producing pelvic pain. The studies report a significant correlation between caffeine consumption and development of pains like headache and pelvic pain (48).

A few study limitations should be mentioned. First, the cross-sectional nature of our study prevents any conclusion regarding causality. Prospective cohort studies are a more reliable way of determining casual relation between various risk factors and dysmenorrhea. Second, it was performed in a single university; therefore, the sample may not be representative of all Iranian female students. Third, data collection has been performed by self-report using questionnaires that have resulted in underreporting of the conditions. Future research by using alternative methods such as interviews might obtain a more detailed and complete view with that regard, particularly about alexithymia. Finally, as the study was the first work that revealed that high alexithymia in women with dysmenorrhea, further researches are needed to determine the extent of the associations between predictors and dysmenorrhea. In addition, further studies are needed to explain the role of cultural variables in the association between social support, neurotic personality, and dysmenorrhea. Although this study had some weakness points such as cross-sectional study and self-report symptoms, it had strong points that include being the first Iranian psychologic risk factor assessment, assessing large number of risk factor (20 depended variable), and considering psychologic and nonpsychologic risk factor simultaneously.

In conclusion, our results showed that psychologic factors such as social support, alexithymia, and neurotic personality are the important risk factors for primary dysmenorrhea. In addition, family history of dysmenorrhea, long menstrual bleeding ≥ 7 days, and coffee consumption are the others important risk factors. This study proposes to consider psychologic risk factors in addition to medical and environmental risk factors to evaluation and treatment of primary dysmenorrhea.
